# Pre-treatment with IL2 gene therapy alleviates *Staphylococcus aureus* arthritis in mice

**DOI:** 10.1186/s12879-020-4880-8

**Published:** 2020-02-28

**Authors:** Berglind Bergmann, Ying Fei, Pernilla Jirholt, Zhicheng Hu, Maria Bergquist, Abukar Ali, Catharina Lindholm, Olov Ekwall, Guillaume Churlaud, David Klatzmann, Tao Jin, Inger Gjertsson

**Affiliations:** 1 Department of Rheumatology and Inflammation Research, Institute of Medicine, Sahlgrenska Academy University of Gothenburg, Gothenburg, Sweden; 2 Department of Microbiology and Immunology, GuiZhou Medical University, Guiyang, People’s Republic of China; 3 Present address: Clinical Sample Scientist at Astrazeneca, Mölndal, Sweden; 4 Present address: Research Physician at Astrazeneca, Mölndal, Sweden; 5 Department of Pediatrics, Institute of Clinical Sciences, Sahlgrenska Academy University of Gothenburg, Gothenburg, Sweden; 6 AP-HP, Groupe Hospitalier Pitié-Salpêtrière, Biotherapy (CIC-BTi) and Inflammation-Immunopathology-Biotherapy Department (i2B), Paris, France; 7 Sorbonne Université, INSERM, Immunology-Immunopathology-Immunotherapy (i3), Paris, France

**Keywords:** T regulatory cells, Tregs, IL2, *S. aureus*, Arthritis, Mice

## Abstract

**Background:**

*Staphylococcus aureus* (*S. aureus*) arthritis is one of the most detrimental joint diseases known and leads to severe joint destruction within days. We hypothesized that the provision of auxiliary immunoregulation via an expanded compartment of T regulatory cells (Tregs) could dampen detrimental aspects of the host immune response whilst preserving its protective nature. Administration of low-dose interleukin 2 (IL2) preferentially expands Tregs, and is being studied as a treatment choice in several autoimmune conditions. We aimed to evaluate the role of IL2 and Tregs in septic arthritis using a well-established mouse model of haematogenously spred *S. aureus* arthritis.

**Methods:**

C57BL/6 or NMRI mice we intravenously (iv) injected with a defined dose of *S. aureus* LS-1 or Newman and the role of IL2 and Tregs were assessed by the following approaches: IL2 was endogenously delivered by intraperitoneal injection of a recombinant adeno-associated virus vector (rAAV) before iv *S. aureus* inoculation; Tregs were depleted before and during *S. aureus* arthritis using antiCD25 antibodies; Tregs were adoptively transferred before induction of *S. aureus* arthritis and finally, recombinant IL2 was used as a treatment starting day 3 after *S. aureus* injection*.* Studied outcomes included survival, weight change, bacterial clearance, and joint damage.

**Results:**

Expansion of Tregs induced by IL2 gene therapy prior to disease onset does not compromise host resistance to *S. aureus* infection, as the increased proportions of Tregs reduced the arthritis severity as well as the systemic inflammatory response, while simultaneously preserving the host’s ability to clear the infection.

**Conclusions:**

Pre-treatment with IL2 gene therapy dampens detrimental immune responses but preserves appropriate host defense, which alleviates *S. aureus* septic arthritis in a mouse model.

## Introduction

*Staphylococcus aureus* (*S. aureus*) is one of the pathogens most commonly causing life-threatening conditions, especially in individuals with underlying health conditions; this is especially relevant to present-day society with its ageing population, its increased burden of chronic diseases such as cancers and autoimmune diseases, and the chemotherapeutic and immunosuppressive treatments that accompany them [1]. One particularly severe manifestation is *S. aureus* arthritis, which is characterized by a rapid destruction of the joints, often followed by permanent and disabling articular damage despite appropriate antibiotic therapy [2].

Innate immunity has been shown to be protective during *S. aureus* arthritis [3–5], but the role of the adaptive immune response is less clear. Clonal expansion of T lymphocytes plays a significant role in the induction of *S. aureus* arthritis and CD4^+^ T cells are considered to be pathogenic in this disease [6]*. S. aureus* infection induces memory T cells against extracellular staphylococcal antigens, and the presence of memory T cells might influence the course of infection [7], but at the same time, the staphylococci are apparently able to dampen T cell responses using several strategies to promote their own survival [8]. One of these is to limit bacterial clearance by expanding T regulatory cells (Tregs) and myeloid derived suppressor cells [9]. The part played by Tregs in *S. aureus* arthritis is unclear. Tregs are defined by the expression of CD4, CD25 and their essential transcription factor, Forkhead box protein 3 (FoxP3), and they have been implicated in the regulation of autoimmune diseases [10–14]. In autoimmune arthritis, Tregs suppress arthritis development and prevent osteoclast activation, thus reducing subsequent bone erosion [15]. Tregs constitutively express the IL2 receptor (IL2R) and although they do not produce IL2 themselves, they are dependent on IL2 for their peripheral homeostasis and maintenance [16, 17]. Administration of low-dose IL2 tips the balance between Tregs and T effector cells (Teffs) towards Tregs [18] showing great promise for the treatment of autoimmune disorders [19–23]. Despite these successes, little is known of how the presence of low-dose IL2 and the consequent expansion of Tregs could affect beneficial effector immune responses when patients receiving the treatment develop acute bacterial infections, such as *S. aureus* arthritis. We hypothesize that although the staphylococci themselves upregulate Tregs during the infection to evade the host immune response [8, 24], a further expansion of Tregs could ameliorate the excessive inflammatory response that is responsible for joint damage and the subsequent detrimental sequelae of this disease. As these studies are very difficult to perform in humans, the aim of this study was to determine whether IL2 and its impact on Tregs influence the course of *S. aureus* arthritis with respect to survival, bacterial clearance and joint damage in our well-established mouse model of hematogenously spread *S. aureus* septic arthritis [25].

## Methods

### Mice strains, ethics statement for animal experiments and randomization

Naval Medical Research Institute (NMRI) and 6–8 weeks old wildtype C57BL/6 mice of both sexes were obtained from Charles River Laboratories (Sulzfeld, Germany) and Scanbur (Sollentuna, Sweden), respectively. Mice were maintained under standard conditions of temperature and light and fed laboratory chow and water ad libitum, at the SPF animal facility of the Department of Rheumatology and Inflammation Research at the University of Gothenburg, Sweden. Mice were hosted up to 10 animals per cage, and both actively treated animals and controls were mixed in the same cage. Mice were allocated to active or control group randomly before the experiments started and assessed by the examiners in a blind manner. All procedures with living mice were performed in the animal house laboratory. Experiments were approved by the Animal Research Ethical Committee of Gothenburg and animal experimentation guidelines were followed strictly (38–2016).

### Recombinant adeno-associated viral vector generation and administration

Recombinant adeno-associated viral vectors (rAVV) of serotype 8 were generated by triple transfection of human embryonic kidney 293 T cells (293 T/17 SF [HEK 293 T/17 SF ATCC® ACS-4500)] [26]. The transgenes luciferase (LUC) and murine IL2 were used and driven by the hybrid cytomegalovirus enhancer/chicken beta-actin constitutive promoter. Mice were injected once intraperitoneally (ip) 19 days prior to bacterial inoculation (day 0) with 10^10^ viral genomes (vg) of rAAV in a total volume of 100 μl of phosphate-buffered saline (PBS).

### Bacterial strain and doses

Two strains of *S. aureus* were used: the clinically isolated LS-1 strain, that produces Toxic Shock Syndrome Toxin 1 (TSST-1), and the laboratory strain, Newman was used in one experiment. The bacterial doses were adjusted to the mouse strain and the purpose of the experiments [25, 27] are summarized in Table 1.

**Table 1:** Summary of mouse experiments

Experiment	Controls	Treatment regimen	*S. aureus* strain	Bacterial dose (CFU/mouse)	Mice
Infected mice	Healthy mice	No treatment	LS-1	1 × 10^7^	NMRI *N* = 44, one experiment
rAAV-IL2	rAAV-LUC	10^10^ vg at day −19 prior to bacterial inoculation	LS-1	5 × 10^7^	C57BL/6 *N* = 60, two experiments
Adoptive transfer	PBS	0.9 × 10^6^ CD4^+^CD25^+^ cells at day −2 prior to bacterial inoculation	LS-1	5 × 10^7^	C57BL/6 *N* = 12, one experiment
IL2 treatment	PBS	Daily injections, starting at day 3 post bacterial inoculation	LS-1	5 × 10^7^	C57BL/6 *N* = 18, two experiments
Anti-CD25 antibody treatment	Rat IgG1 antibody	One injection at day −3 prior bacterial inoculation and at day 3 post bacterial inoculation	(1) LS-1 (2) Newman	(1) 3.5 × 10^7^ (2) 6.5 × 10^6^	(1) C57BL/6 *N* = 20, one experiment (2) NMRI *N* = 28, one experiment

### Mouse model of *S. aureus* arthritis

Table 1 summarizes the experiments, numbers of mice and of colony forming units (CFU) inoculated per mouse. In total 182 mice were used, including 72 NMRI mice and 110 C57BL/6 mice. Pre-made batches of bacteria were thawed, washed and diluted to the desired concentration. To avoid adverse events, the number of inoculated CFU was carefully assessed with respect to bacterial strain and mouse strain. The food was placed at the bottom of the cage and the water tanks had extra long tips to aoid inflicting more pain to inflamed paws when feeding. Mice were inoculated via the tail vein with 0.2 ml of *S. aureus*. Thereafter, mice were weighed regularly by observers blinded to their treatment group (P.J., I.G., MN.N. and T.J.). On indicated days the mice were anesthetized with ketamine hydrochloride (Pfizer AB, Sweden) and metedomidine (Orion Pharma, Finland) and then sacrificed by cervical dislocation. Hereafter, kidneys were obtained for assessment of bacterial persistence, limbs for radiological and histological evaluations, and spleens, lymph nodes and blood were used for flow cytometry and cytokine analysis.

### Adoptive transfer of Tregs

To expand the Treg compartment of naïve mice sufficiently to provide for the harvesting of Tregs for adoptive transfer, C57BL/6 donor mice (*n* = 10) were injected once ip with 10^10^ vg rAAV-IL2 19 days prior to the bacterial inoculation of experimental mice on day 0. Two days prior to bacterial inoculation, spleens were harvested from the donor mice and CD4^+^CD25^+^ Tregs were isolated with Dynabeads FlowComp Mouse CD4^+^CD25^+^ Treg cells kit (Invitrogen) according to the manufacturer’s instructions. On day 0, 0.9 × 10^6^ CD4^+^CD25^+^ cells in a total volume of 200 μl PBS were transferred iv into recipient (experimental) C57BL/6 mice (*n* = 4), and recipient control mice (*n* = 8) were injected with an equal volume of PBS. Also on day 0, all recipient mice were inoculated iv with the LS-1 strain of *S. aureus*; the experiment was terminated 11 days later, when mice were sacrified for blood and tissue analysis.

### Anti-CD25 antibody treatment

Three days before and 3 days after bacterial inoculation, C57BL/6 mice (*n* = 10) or NMRI mice (*n* = 10) were treated with an ip injection of 500 μg rat anti-mouse CD25 monoclonal antibody (clone: PC.61·5, rIgG1; BioXcell, West Lebanon, NH, USA) in a total volume of 100 μl of PBS. Control C57BL/6 (*n* = 10) or NMRI mice (*n* = 10) treated likewise with rat IgG1 mAb (BioXcell), 500 μg/mouse, served as negative controls.

### IL2 treatment

IL2 treatment (recombinant human IL2, rhIL2, Proleukin®, Novartis) was started on day 3 after bacterial inoculation on day 0. C57BL/6 mice (*n* = 9) were treated with ip injections of 25.000 IU/day/mouse [28] of rhIL2 in a total volume of 100 μl of phosphate-buffered saline (PBS) until the end of the experiment on day 10. Mice in the control group (*n* = 9) were injected with PBS.

### Clinical evaluation of *S. aureus* arthritis

Observers blinded to the treatment groups visually inspected the wrists, ankles, fingers and toes of each mouse during the course of the infection [27]. Arthritis was defined as erythema and/or joint swelling. To evaluate the severity of arthritis, a clinical scoring system from 0 to 3 was used for each paw, as described before [27, 29]. Since the signs of arthritis in deeper joints (e.g. knee and elbow joints) cannot be evaluated clinically, micro-computed tomography (μ-CT) or histopathology of joints was used as necessary for further confirmation of arthritic pathology.

### Histology of inflamed joints

Joints were fixed, decalcified and paraffin embedded. Tissue sections from fore- and hind paws were cut, deparaffinized and stained with hematoxylin-eosin (Histolab Products AB, Gothenburg, Sweden). Stained slides were coded and evaluated by two observers blinded to the treatment groups (B.B. and I.G.). The evaluation was based on inflammatory cell accumulation in synovial tissue (synovitis) and bone erosion. The degrees of synovitis and bone erosion were scored on a scale of 0 to 3 for every joint of finger/toes, wrists/ankles, elbows and knee. For comparison between individuals, the total score/mouse was divided by the number of joints evaluated.

### Microcomputed tomography (μCT)

Joints were fixed in 4% formaldehyde for 3 days and then transferred to PBS for 24 h. Afterwards all 4 limbs were scanned and reconstructed into a three-dimensional structure with Skyscan1176 micro-CT (Bruker, Antwerp, Belgium), as previously described [30]. After reconstruction, the 3D structures of each joint were assessed by two observers blinded to the treatment groups (T.J. and Y.F) using a scoring system from 0 to 3 (0, healthy joint; 1, mild bone destruction; 2, moderate bone destruction; 3, marked bone destruction).

### Bacterial clearance

The host’s ability to clear the *S. aureus* bacteria was measured as the CFU/kidney pair. Extra-articular spread of bacteria in the mouse model is predominantly to the kidneys and spleen and since culturing bacteria from murine joints is challenging in practice, we used the kidney cultures to reflect the bacterial clearance. Kidneys were aseptically dissected, kept on ice, homogenized, serially diluted in PBS and spread on blood agar plates. After 24 h of incubation at 37 °C, the number of CFU/kidney pair was determined.

### Cell preparation and flow cytometry

Single cell suspensions were prepared from blood, regional lymph nodes, spleens and kidneys, and were pre-incubated with Fc-block (BD Biosciences). The antibodies used are summarized in Table 2. Analyses were performed on a FACSCanto II equipped with FACSDiva software (BD Bioscience), and with the FlowJo software (Tree Star Inc.). The gating strategies were based on fluorochrome minus one (FMO) setting, when needed.

**Table 2:** Antibodies used for flow cytometry

Mouse Antigen	Clone	Source
CD4	L3T4	BD Biosciences
CD25	PC61	Biolegend
Foxp3	NRRF-30	eBioscience
CD19	ID3	eBioscience
NK1.1	PK136	BD Biosciences
TCRβ	H57–597	eBioscience

### Cytokine analysis

Th1/Th2 cytokines (GM-CSF, IFNγ, IL1α, IL10, IL17A, IL2, IL4, IL5, IL6 and TNF) were analyzed in serum from rAAV treated mice using the Mouse Th1/Th2 10-plex Ready-to-Use FlowCytomix Multiplex kit (eBioscience) according to the manufacturer’s recommendations. Samples were analysed with a FACS Canto II.

### Statistical analysis

Statistical analyses were performed using GraphPad Prism (GraphPad, La Jolla, CA). Statistical differences between groups were calculated using the Mann-Whitney U test, Student’s *t* test and one-way ANOVA. Kaplan-Meier survival plots were prepared and the log-rank test was used for comparison between the two survival curves. *P* < 0.05 was considered statistically significant.

All data generated or analysed during this study are included in this published article.

## Results

### The frequencies of CD4^+^CD25^+^FoxP3^+^ Tregs in blood, spleen and lymph nodes increased during the natural course of *S. aureus* arthritis

**Fig. 1:**
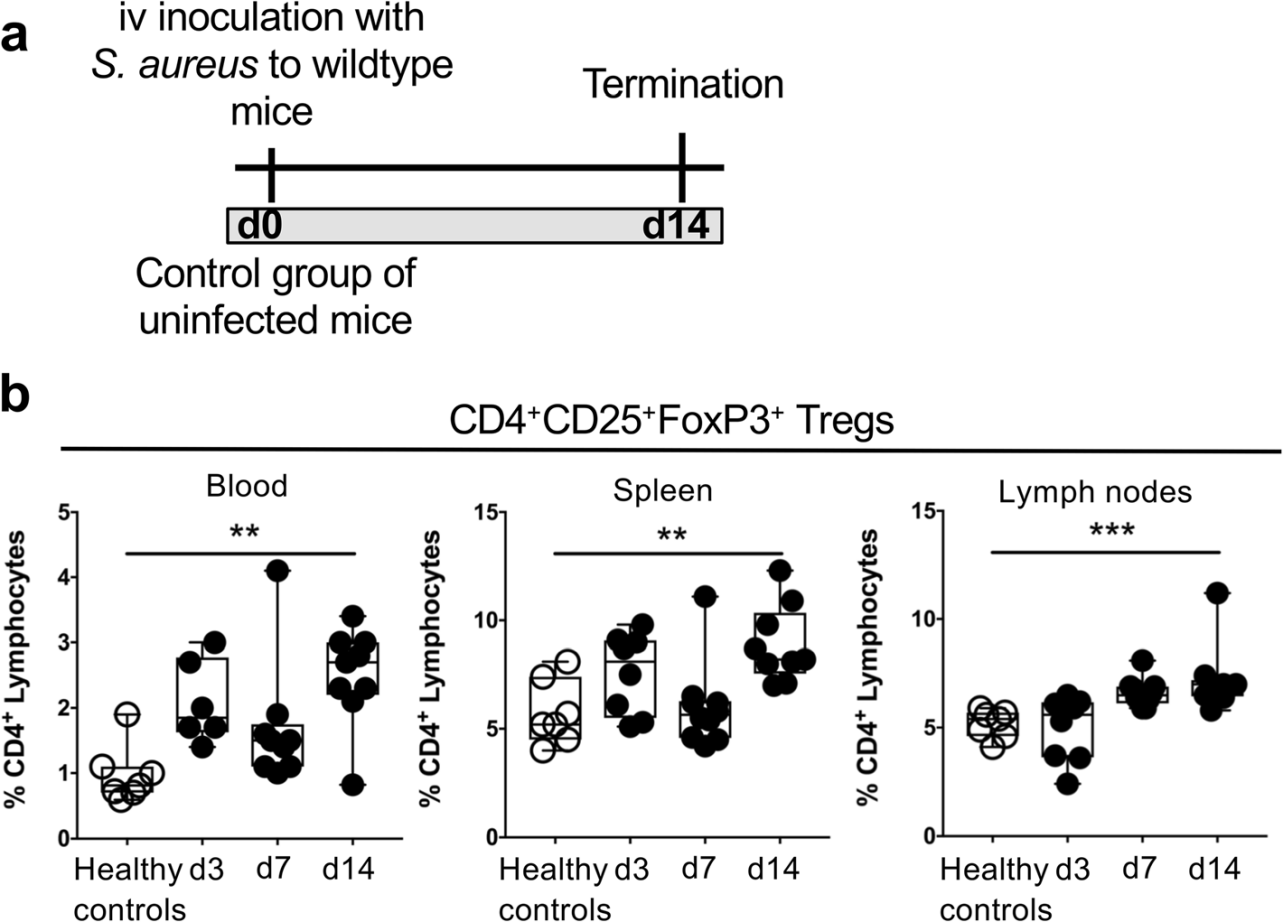
Tregs in mice with *S. aureus* arthritis. **a**, Experimental set-up for the evaluation of Tregs proportions during the natural course of *S. aureus* arthritis. **b***,* Tregs expressed as frequency of CD25^+^FoxP3^+^ of the CD4^+^ lymphocytes in blood, spleen and lymph nodes in healthy uninfected mice and in mice with *S. aureus* arthritis. In panel B, data are shown as median, whiskers = min to max. Statistical calculations were made using the Mann-Whitney U-test and one-way ANOVA, ** *P* < 0.01, ***P < 0.001

To study the physiological changes in Treg proportions during the natural course of *S. aureus* arthritis, mice were inoculated iv with *S. aureus* at day 0 and healthy uninfected mice were used as controls (Fig. 1a). The frequencies of CD4^+^CD25^+^FoxP3^+^ Tregs in blood, spleen and lymph nodes of mice with *S. aureus* arthritis, from day 3 to 14 after bacterial inoculation, were higher than those in healthy control mice (Fig. 1b).

### Persistent IL2 release by rAAV-IL2 enhanced bacterial elimination and reduced systemic inflammation as well as joint damage in *S. aureus* arthritis

**Fig. 2:**
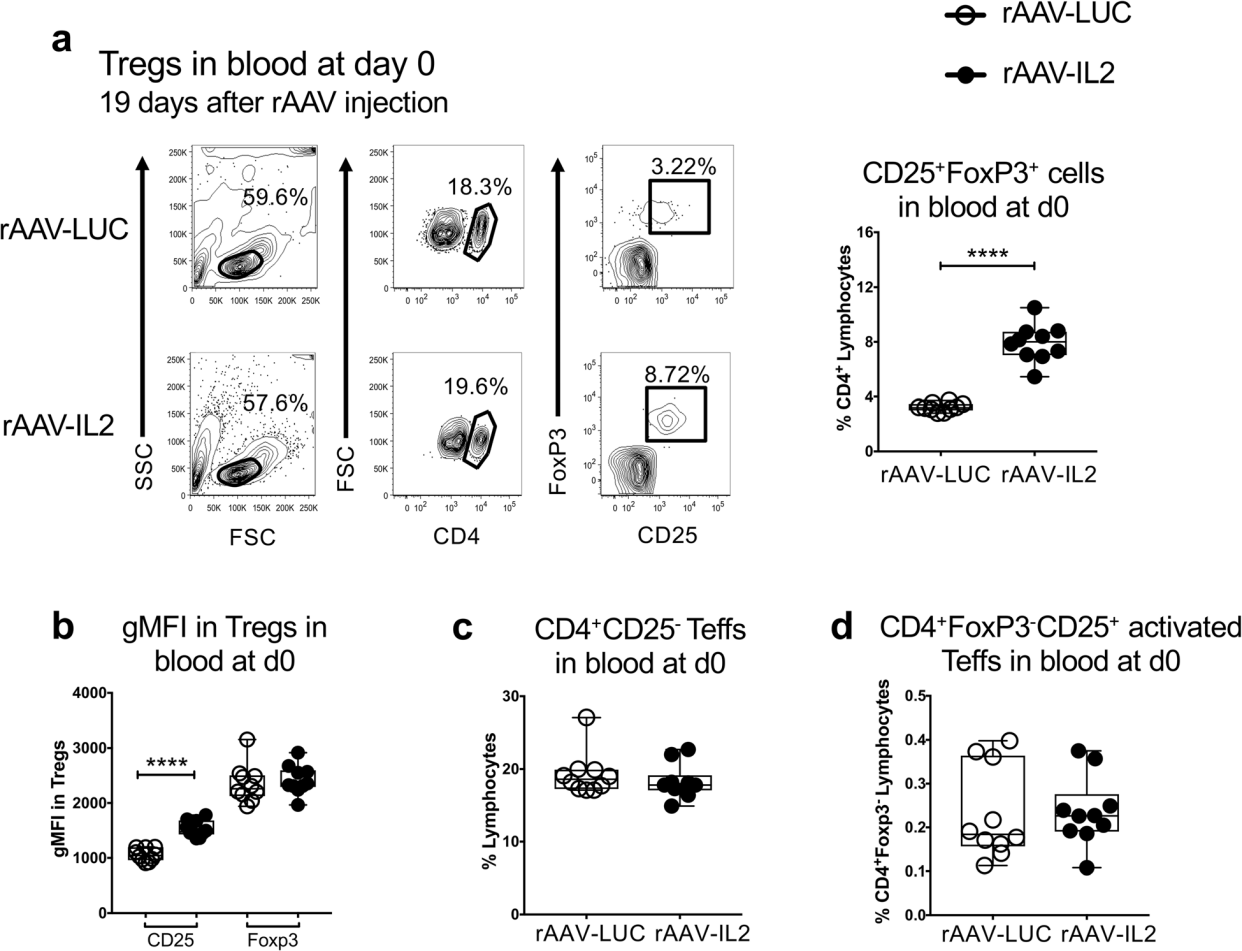
Frequencies and T cell phenotypes at day 0, i.e. 19 days after inoculation of the rAAV-LUC/IL2 vectors. **a**, Gating strategies and frequency of CD25^+^FoxP3^+^ out of the CD4^+^ lymphocytes in blood at day 0. **b***,* Protein expression levels of CD25 and FoxP3 (measured as geometric mean fluorescence (gMFI)), in Tregs in rAAV-LUC and rAAV-IL2 treated animals. **c**, The frequency of CD4^+^CD25^−^ Teff cells at day 0. **d**, The frequency of CD4^+^CD25^+^FoxP3^+^ activated Teff cells at day 0. Data are shown as median, whiskers = min to max. Statistical calculations were made using Mann-Whitney U-test, ****P < 0.0001

To expand the Treg compartment in experimental mice, they were injected once intraperitoneally (ip) with recombinant adeno-associated virus vector (rAAV) encoding IL2 or luciferase (LUC) at day − 19. At day 0, i.e. 19 days later the proportion of CD4^+^CD25^+^FoxP3^+^ Tregs in the peripheral blood of IL2 treated mice was significantly increased compared to controls, as was the level of expression on the Tregs of CD25 (measured as geometric mean fluorescence (gMFI)), but not of FoxP3 (Fig. 2a-b). The rAAV-mediated IL2 release did not affect the frequencies of CD4^+^CD25^−^ Teffs or CD4^+^CD25^+^FoxP3^−^ activated Teffs (Fig. 2c-d). These results are in agreement with previous results [31], and show that continuous rAAV-mediated release of murine IL2 in this fashion increases and activates Tregs, without affecting Teffs.

**Fig. 3:**
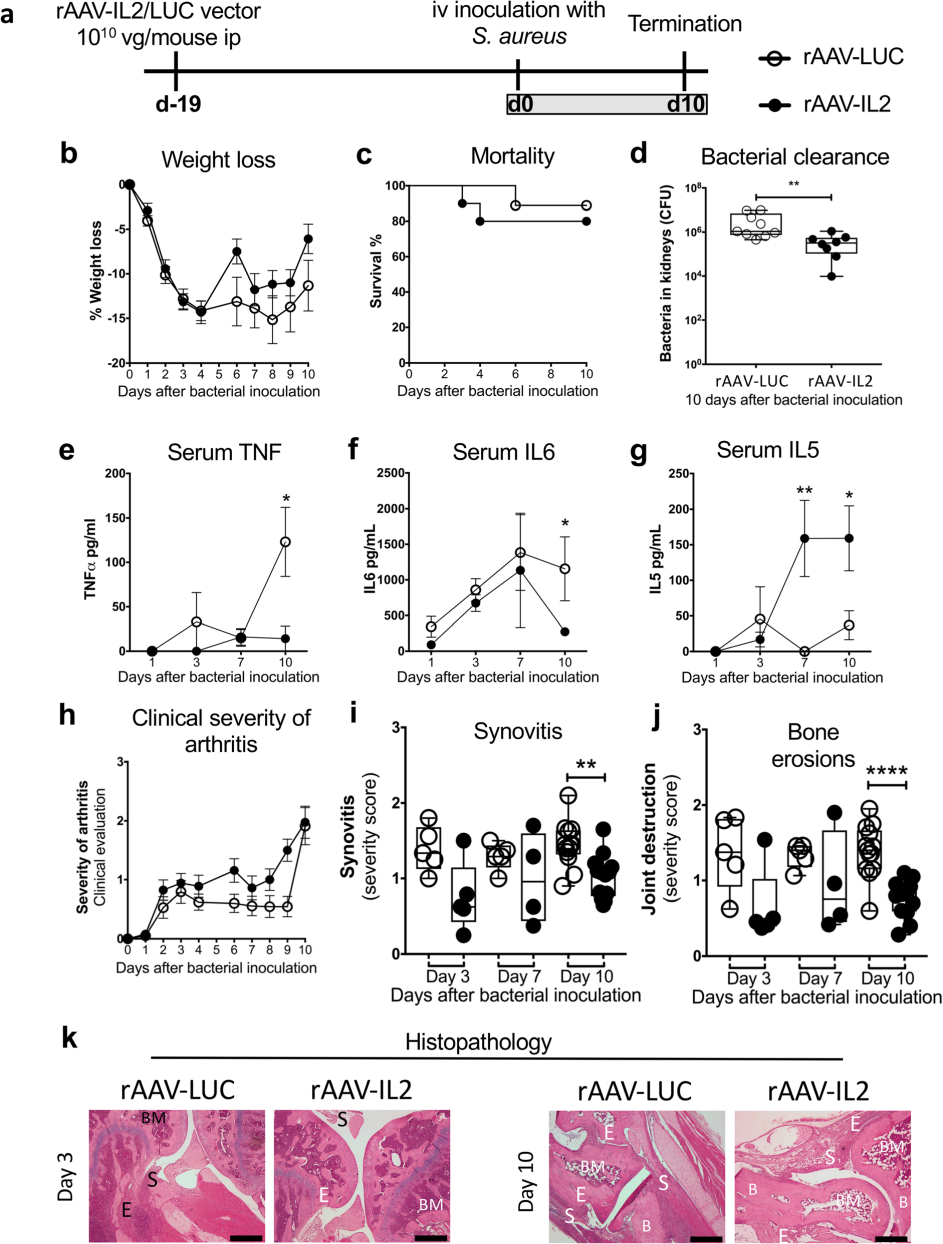
The clinical development of *S. aureus* arthritis in rAAV-LUC/IL2 mice. **a**, Experimental set up. **b**, Weight loss. **c***,* Mortality. **d***,* Bacterial clearance in the kidneys. **e-g***,* Serum protein levels of **e***,* TNF, **f**, IL6 and **g**, IL5. *H,* Clinical development of arthritis. **i-k***,* The severity of joint inflammation, or synovitis, and bone erosions was evaluated by making a histological scoring of **i**, synovitis and **j**, bone erosions. **k***,* Representative H&E stained sections of inflamed joints on day 3 and 10 after bacterial inoculation. In panels **b** and **d-h**, bars show the mean ± standard error of the mean (SEM). In panels **i** and **j**, data are shown as median, whiskers = min to max. Statistical calculations were made using the Mann-Whitney U-test. Kaplan-Meier survival plots were prepared and the log-rank test was used for comparison between the two survival curves. *P < 0.05, **P < 0.01. ****P < 0.0001. Scale bars, 100 μm. Abbreviations: B, bone; BM, bone marrow; BD, bone destruction; C, cartilage; E, bone erosions; S, Synovitis

To establish *S. aureus* arthritis in the mice with persistent IL2 release, they were intravenously (iv) inoculated with *S. aureus* on day 0 (Fig. 3a). Weight loss, which is used as a measurement of morbidity in this mouse model, and mortality were not affected by the rAAV-IL2 treatment (Fig. 3b-c). At day 10 after bacterial inoculation, the host’s ability to clear the bacteria was increased in the rAAV-IL2-treated group compared to the control group (Fig. 3d). Systemic levels of proinflammatory cytokines are elevated in *S. aureus* arthritis and correlate well with disease severity and morbidity [32, 33]. Serum levels of TNF and IL6 were significantly decreased in the rAAV-IL2 group compared to controls 10 days after bacterial inoculation (Fig. 3e-f*)*. Serum levels of IL5 were significantly increased in the rAAV-IL2 group compared with controls, at both 7 and 10 days after bacterial inoculation (Fig. 3g). Serum levels of GM-CSF, IFNγ, IL1α, IL10, IL17A, IL2 and IL4 were all below detection limits. These results show that relative to controls, mice with enhanced Treg compartments at bacterial inoculation and persistent IL2 release during the course of *S. aureus* arthritis had reduced systemic inflammatory responses and improved bacterial clearance 10 days after inoculation, without adverse effects on their mortality or morbidity.

We hypothesized that increased immunoregulation would reduce the severe and irreversible joint damage in *S. aureus* arthritis, as it is the bone erosivity that gives rise to the sequelae, and it is the excessive immune response in this infection that is responsible for the joint damage. To test this prediction, we evaluated clinical and histopathological signs of arthritis. There were no differences between the groups with respect to severity of clinically evaluated arthritis during the course of infection (Fig. 3h). However, histopathological evaluations of the joints 10 days after bacterial inoculation showed that the rAAV-IL2-treated mice had a significant reduction in the severity of synovitis (Fig. 3i and k) and bone erosion (Fig. 3j-k), compared to controls.

### Persistent IL2 release by rAAV-IL2 led to an increase in the frequencies of Tregs, B, NK and NKT cells prior to bacterial inoculation but only Treg frequency remained elevated during *S. aureus* arthritis

**Fig. 4:**
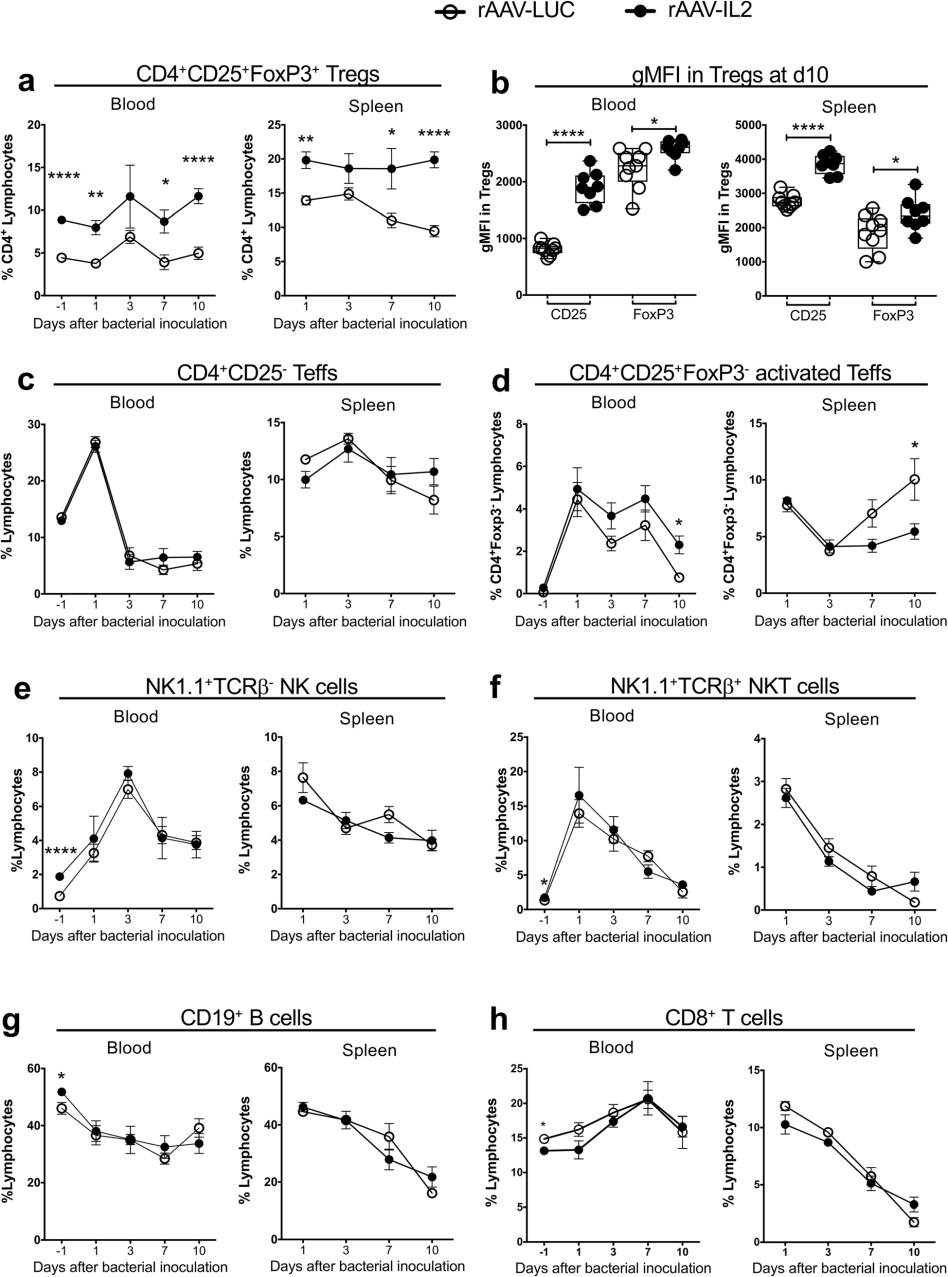
Effects of persistent IL2 production induced using an IL2-producing rAAV vector in *S. aureus* arthritis. **a-b***,* Characteristics of CD4^+^CD25^+^FoxP3^+^ Tregs from peripheral blood and spleen **a***,* Frequency measured during the course of *S. aureus* arthritis. **b** Protein expression levels of CD25 and FoxP3 (measured as geometric mean fluorescence (gMFI)), at day 10 after bacterial inoculation. **c-g**, Frequencies of other lymphocytes measured during the course of *S. aureus* arthritis in blood and spleen. **c**, CD4^+^CD25^−^ Teffs **d**, CD4^+^CD25^+^FoxP3^−^ activated Teffs. **e**, NK1.1^+^TCRβ^−^ NK **f**, NK1.1^+^TCRβ^+^ NKT **g**, B cells **h**, CD8^+^ T cells. In panels **a** and **c-h**, bars show the mean ± standard error of the mean (SEM). In panel **b** data is shown as median, whiskers = min to max. Statistical calculations were made using Mann-Whitney U-test, *P < 0.05, ****P < 0.0001

To elucidate the mechanism behind the beneficial effects of rAAV-induced IL2 release in *S. aureus* arthritis, we analyzed the composition of different immune cell types from mice receiving rAAV-IL2 or LUC during the course of *S. aureus* arthritis. The frequency of CD4^+^CD25^+^FoxP3^+^ Tregs in peripheral blood and spleen was significantly higher in the rAAV-IL2 treated group than in the control group throughout the infection (Fig. 4a). At termination, i.e. 10 days after bacterial inoculation, the expression levels of CD25 and FoxP3 on Tregs in both blood and spleen of treated mice were also significantly higher than on those of controls (Fig. 4b). The frequency of CD4^+^CD25^−^ Teffs in blood and spleen throughout the infection was not affected by rAAV-IL2 treatment (Fig. 4c). At day 10, the frequency of CD4^+^CD25^+^FoxP3^−^ activated Teffs in the rAAV-IL2 group was significantly increased in blood but decreased in spleen, compared to controls (Fig. 4d). Otherwise, the frequencies of CD4^+^CD25^+^FoxP3^−^ activated Teffs were similar in both groups. Thus, i.p. injection of rAAV-IL2 expanded and activated Tregs throughout the infection without major impact on Teffs.

Because Tregs are not the only target of IL2 action, we assessed its’ influence on other cell populations. The frequencies of NK cells, NKT, B cells and CD8^+^ T cells in peripheral blood were significantly elevated in treated mice compared to controls 19 days after rAAV-IL2 injection (Fig. 4e-h), but this difference disappeared following bacterial inoculation (day 1 and onwards). For the remaining duration of the experiment, there were no differences between treatment groups with respect to the proportions or absolute numbers of NK, NKT, B cells or CD8^+^ T cells in peripheral blood and in the spleen (Fig. 4e-h).

### Depletion of CD25^+^ cells aggravated *S. aureus* arthritis

**Fig. 5:**
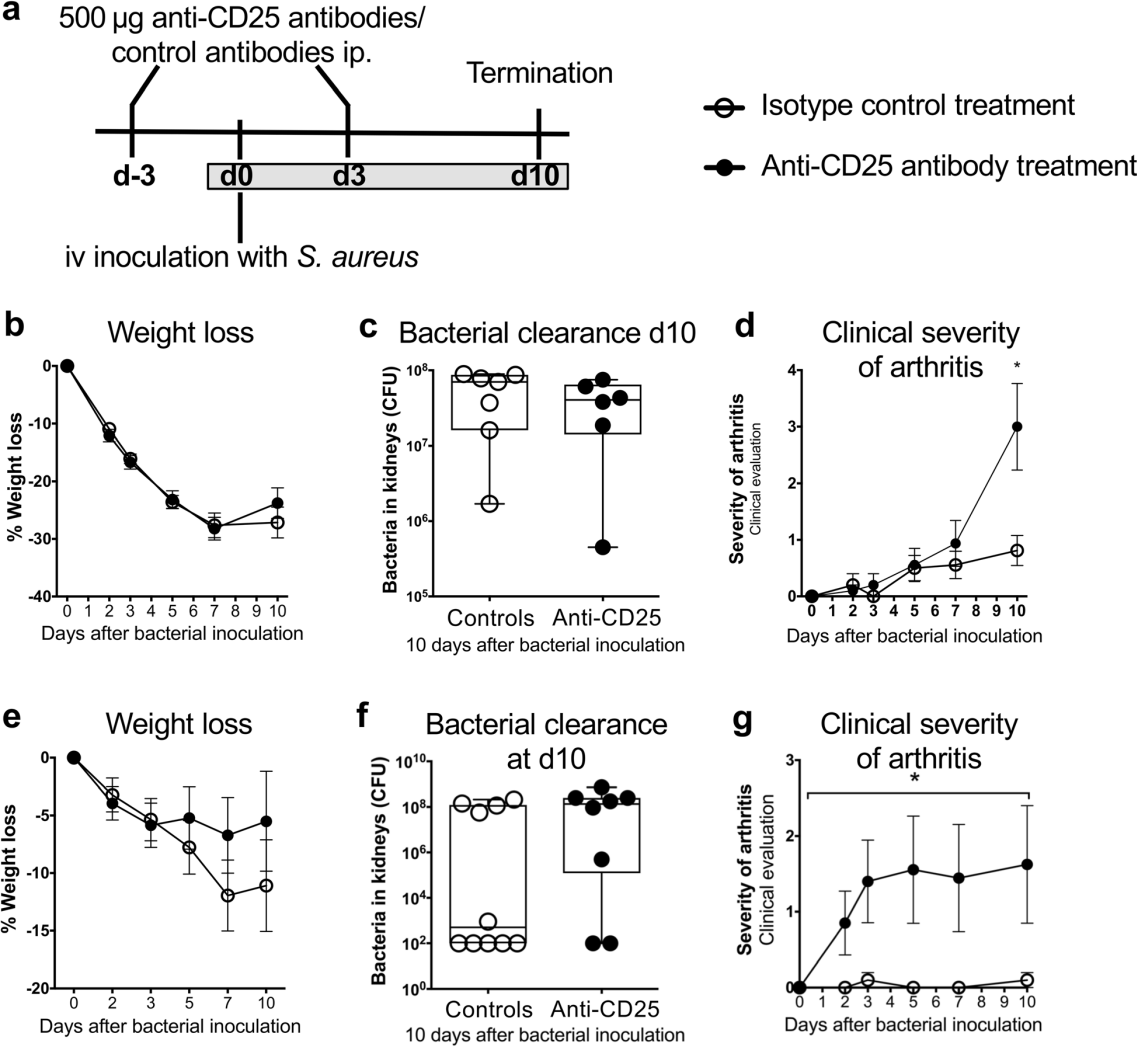
Depletion of CD25^+^ Tregs. **a**, Experimental set-up for the depletion of Tregs using anti-CD25 antibodies and isotype control antibodies. **b***,* Weight loss. **c***,* Bacterial clearance in the kidneys. **d***,* Clinical arthritis development. Depletion of CD25^+^ Tregs using anti-CD25 antibodies and isotype control antibodies in NMRI mice: **e***,* Weight loss during the course of *S. aureus* arthritis. **f***,* Bacterial clearance in the kidneys at day 10 after bacterial inoculation. **g***,* Clinical arthritis development. In panels **b**, **d**, **e** and **g**, bars show the mean ± standard error of the mean (SEM). In panels **c**, and **f**, data are shown as median, whiskers = min to max. Statistical calculations were made using the Mann-Whitney U-test and one-way ANOVA, *P < 0.05

As the elevated frequencies of NK and NKT cells achieved prior to infection could provide one explanation for the beneficial effects of rAAV-IL2 treatment on *S. aureus* arthritis, e.g. through improved bacterial killing, we next sought to isolate the Treg-mediated effects. Depletion of CD25^+^ cells with anti-CD25 antibodies has been shown to deplete Tregs, but when implemented in our experimental mice (Fig. 5a), it did not significantly affect weight loss or bacterial clearance at day 10 (Fig. 5b-c). However, C57BL/6 mice depleted of CD25^+^ cells developed a more severe clinical arthritis after intravenous inoculation of the *S. aureus* LS-1 strain (Fig. 5d). In addition, similar results were obtained when the experiment was repeated with NMRI mice. These mice were also depleted of CD25^+^ cells but instead of LS-1, the *S. aureus* Newman strain was used (Fig. 5e-g). Taken together these results suggest that the Treg compartment protects against joint destruction.

### Adoptive transfer of CD4^+^CD25^+^ Tregs in *S. aureus* arthritis

**Fig. 6:**
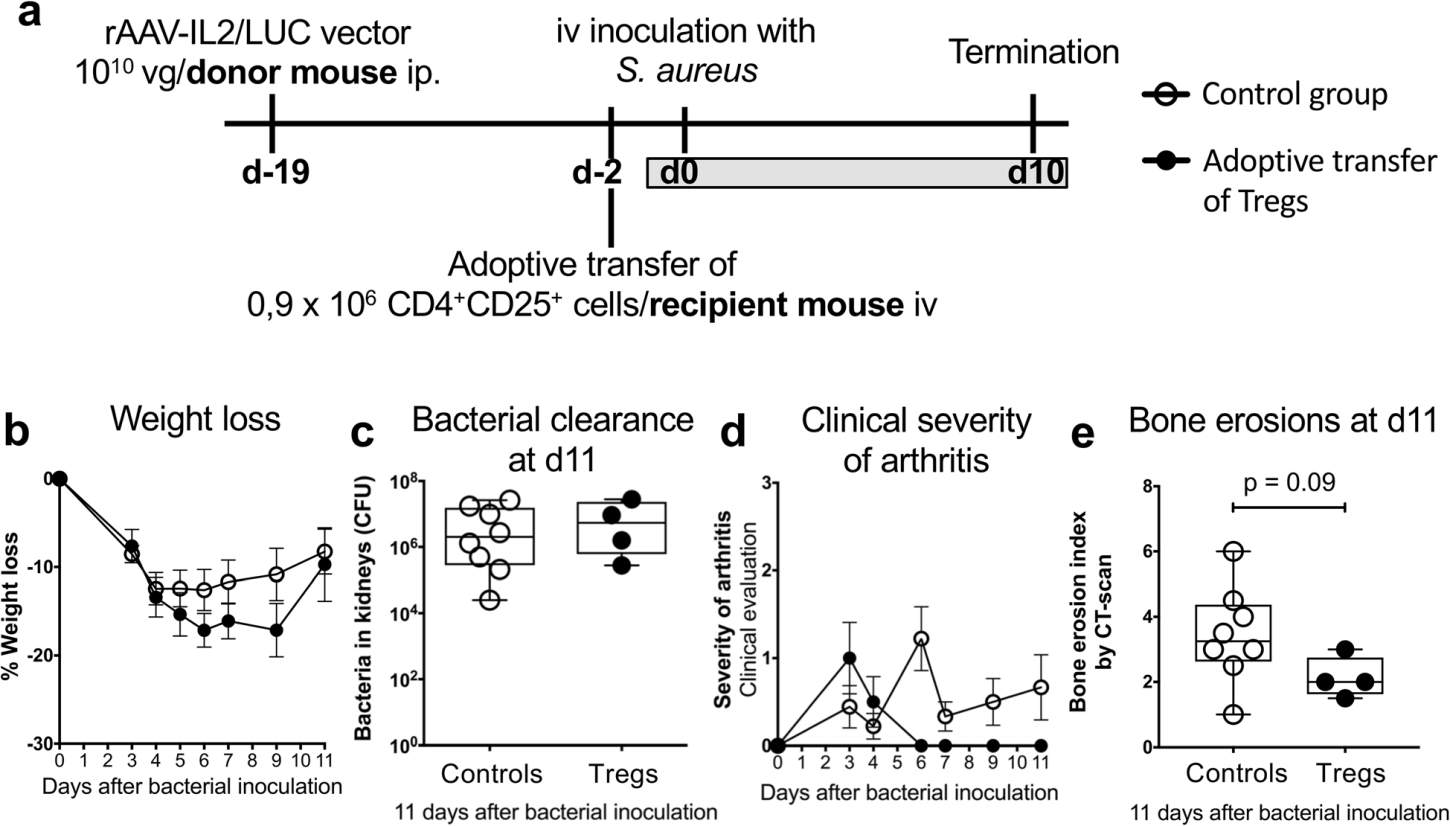
The experimental set-up of adoptive transfer of Tregs in *S. aureus* arthritis. **a**, Experimental set up. **b**, Weight loss. **c**, Bacterial clearance in the kidneys. **D,** Clinical severity of arthritis. **e**, Bone erosions evaluated with micro-CT. In panels **b**, and **d**, bars show the mean ± standard error of the mean (SEM). In panels **c**, and **e**, data are shown as median, whiskers = min to max. Statistical calculations were made using the Mann-Whitney U-test and one-way ANOVA, *P < 0.05

To find out whether adoptive transfer of Tregs is protective in *S. aureus* arthritis, CD4^+^CD25^+^ Tregs were harvested 19 days after rAAV-IL2 injection from donor mice and transferred to recipient mice (Fig. 6a). No differences in weight loss or bacterial clearance were found between the groups at day 11 (Fig. 6b-c). Although mice that had received Tregs tended to have lower scores for clinical arthritis and micro-CT-evaluated bone erosions, the differences were not statistically significant (Fig. 6d-e).

### Recombinant IL2 treatment after onset of *S. aureus* arthritis increased the frequency of Tregs but did not alter arthritis development

**Fig. 7:**
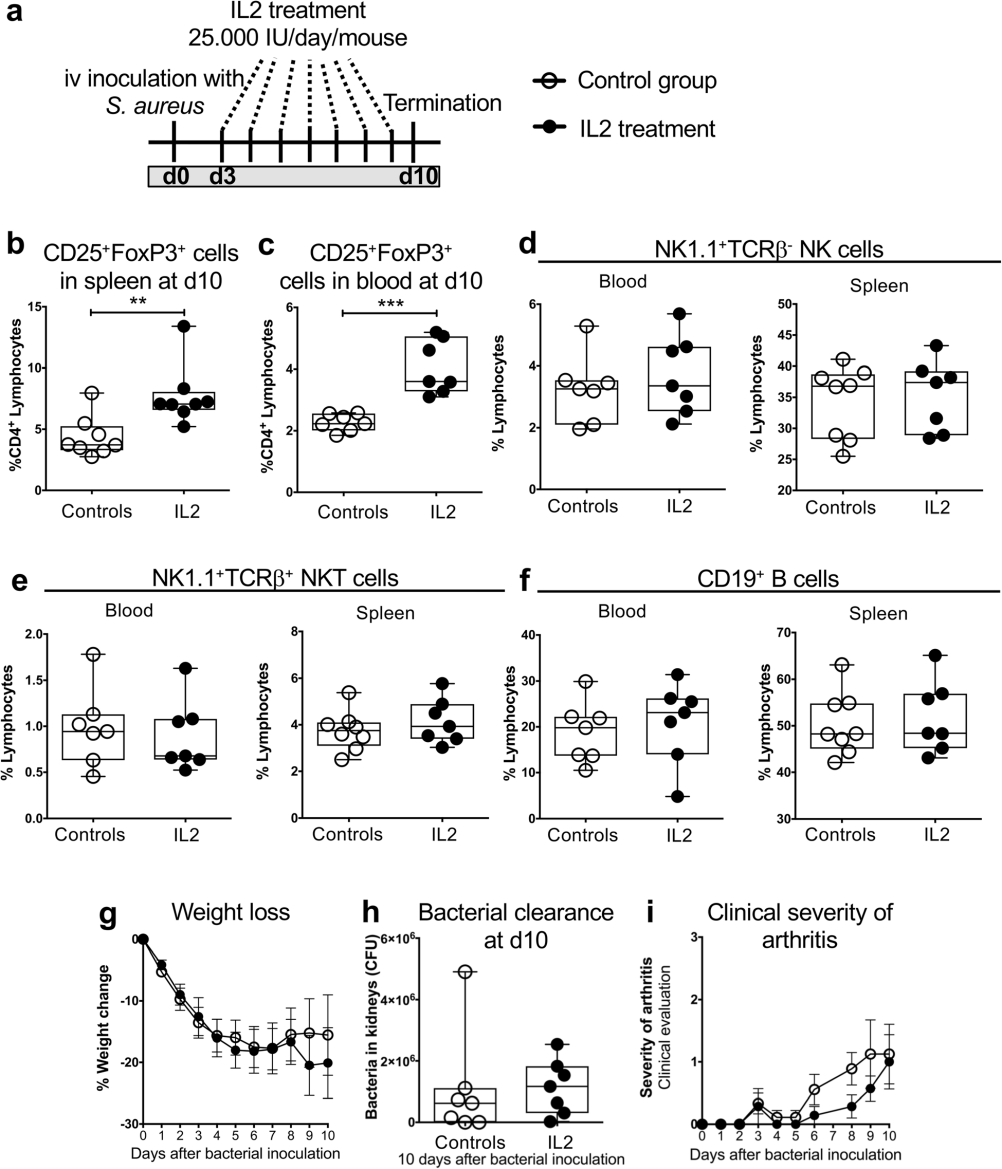
Treatment with rhIL2 in *S. aureus* arthritis. **a***,* Experimental set-up. Frequency of CD25^+^FoxP3^+^ of CD4^+^ lymphocytes in **b**, spleen and **c***,* blood at day 10 after bacterial inoculation. The frequencies of **d**, NK, **e**, NKT and **f**, B cells in blood 10 days after bacterial inoculation in mice after treatment with rhIL2. **g***,* Weight loss. **h***,* Bacterial clearance in the kidneys. **i***,* Clinical severity of arthritis. In panels **b**, **c** and **e**, data are shown as median, whiskers = min to max. In panels **d** and **f**, bars show the mean ± standard error of the mean (SEM). Statistical calculations were made using the Mann-Whitney U-test, **P < 0.01, ***P < 0.01

To investigate whether an expanded Treg compartment at the onset of *S. aureus-*induced arthritis is a prerequisite for the beneficial effects of Tregs on joint damage, or whether IL2 might also be a useful treatment option in septic arthritis, mice were inoculated with *S. aureus* and treatment with exogenous rhIL2 was started at day 3 after infection, when clinical signs of arthritis are evident (Fig. 7a). The proportions of Tregs in spleen and peripheral blood 10 days after bacterial inoculation were significantly increased in the rhIL2-treated animals compared to controls (Fig. 7b-c). However, no differences were detected in the proportions of NK, NKT, or B cells in spleen or blood at termination (Fig. 7d-f). Weight loss, bacterial clearance and clinical severity of arthritis were all similar in both groups (Fig. 7g-i). Thus, it appears that the Treg compartment needs to be expanded before bacterial inoculation to have a beneficial effect on arthritis development.

## Discussion

*S. aureus* arthritis remains an infection with high morbidity and mortality, which has proven difficult to treat effectively due to the conflicting requirements of a strong immune response to ensure bacterial elimination, and concomitant mitigation of the tissue destructive effects of that strong immune response. The results we present here show that the combination of ip injection of rAAV-IL2 and an expanded Treg compartment, present at the onset of *S. aureus* arthritis, is beneficial with respect to joint damage and does not dampen protective host responses.

As others have found [9], we show here a physiological increase in the frequency of CD4^+^CD25^+^FoxP3^+^ Tregs in blood, spleen and lymph nodes during the natural course of *S. aureus* arthritis, which is most likely due to the host’s regulation of the evoked immune response. The rAAV-induced IL2 persistent release that we used here, before and during the course of *S. aureus* arthritis, is known to robustly expand CD4^+^CD25^+^FoxP3^+^ Tregs in both blood and spleen [31]; we have confirmed this effect and found it to be sustained for the duration of the infection, without a major impact on the Teffs. An expanded Treg compartment at bacterial inoculation and persistent IL2 release during the course of *S. aureus* arthritis reduced the systemic inflammatory response and led to an increase in bacterial clearance 10 days after bacterial inoculation, with no adverse effects on mortality or morbidity. We hypothesized that the increased immunoregulation would reduce the severe and irreversible joint damage in *S. aureus* arthritis, as it is the bone erosivity that gives the sequelae, and it is the excessive immune response in this infection that is responsible for the joint damage. In fact, our treatment led to a reduction in both synovitis and bone erosion in the rAAV-IL2-treated mice, compared to controls.

The beneficial effects we observed, however, may not be solely attributable to the Tregs themselves. The rAAV-IL2 mediated IL2 release resulted in a higher frequency relative to controls of NK cells at bacterial inoculation but not later during the infection. NK cells express high levels of the intermediate-affinity CD122, 132 dimeric IL2R and require IL2 for activation and proliferation. Tregs on the other hand, in addition to CD122 and CD132 expression, also constitutively express high levels of CD25 IL2Rα and therefore the high-affinity trimeric IL2R; the resulting enhancement of both their sensitivity to and their consumption of IL2 limits the supply for other cell types [34]. Thus, the failure of the NK cell population to expand following infection could be a result of the preemptive expansion and consequent IL2 depletion by Tregs. It is nonetheless possible that the enlarged NK cell population attributable to IL2 supply before infection contributes to the improved clinical outcome we have observed, as others have found that antibody-mediated depletion of NK cells leads to more profound synovitis and severe joint destruction in *S. aureus* arthritis [35]. Our own previous work, however, has implicated IL15, an absolute requirement for the development of NK cells, in mediating joint damage in *S. aureus* arthritis, indicating that more work is required to permit proper assessment of the NK cell contribution to the current results. Likewise, the relevance of the observed increase in B cell frequencies after rAAV-induced IL2 production is unclear, but the contribution of B cells to host’s defenses in *S. aureus* arthritis is thought to be limited [36].

The increase in Treg frequency and the reduction in bacterial load in the rAAV-IL2 mice relative to controls were accompanied by a significant reduction in serum levels of IL6 and TNF. Together with other cytokines such as IL17, both IL6 and TNF are known to induce the RANKL expression that subsequently enhances osteoclastogenesis and bone destruction, and are thus considered pathogenic in *S. aureus* arthritis [37–40]. Lower levels of IL6 and TNF, an expanded Treg but not Teff compartment, and no adverse effects on morbidity and mortality, combine to suggest that it is a decrease in general inflammation that is responsible for lessening joint destruction, to which the reduced frequency of Teffs in spleen might contribute. It might seem counterintuitive that the bacterial clearance is improved during immunosuppression, but indeed the presence of IL-10 improves bacterial clearance and reduces serum levels of TNF [41]. It is also possible that the increased levels of IL5, which probably are due to both the infection [42] and the increased levels of IL2 [21], could contribute to an improved bacterial clearance as IL5 induces the production of eosinophils that could provide a survival benefit in *S. aureus* bacteremia [43].

Depletion of CD25^+^ Tregs significantly aggravated the clinical arthritis. This is in line with our previous study where we showed that treatment with CTLA4-Ig, which inhibits T cell activation and deletes Tregs, aggravated *S. aureus* arthritis [27]. To determine whether the beneficial effects in *S. aureus* arthritis were due to Tregs or other IL2-mediated effects, we adoptively transferred CD4^+^CD25^+^ Tregs and found a clear, although not statistically significant, tendency to a reduction of both the clinical arthritis and bone erosion. Taken together, these results suggest that Tregs are protective in *S. aureus* arthritis, but the effect obtained by enhanced IL2 production is not solely mediated by Tregs.

Our results suggest that rAAV-induced IL2 production significantly enhanced bacterial killing and reduced the inflammation and bone erosion. Could IL2 therefore be a treatment option in patients with *S. aureus* arthritis? Our treatment with rhIL2 in clinically manifest murine *S. aureus* arthritis had no positive effects on the joint damage or bacterial clearance, suggesting that rhIL2 is not efficient as a treatment in *S. aureus* arthritis when infection is established and disease has progressed. In fact, IL2 treatment did expand the CD4^+^CD25^+^FoxP3^+^ compartment, although to a lesser extent than treatment with the rAAV-induced IL2 (around 7% compared to 20% in spleen, respectively), but failed to enhance the proportion of NK cells. We speculate that in order to have a beneficial effect on the outcome of infection, the Treg compartment, and possibly also the NK cell compartment, need to be expanded already at the onset of infection, and that the expanded compartments might need to reach a certain size.

There are several limitations in the present study. Neither the number of Tregs obtained for adoptive transfer or the dose of rIL2 has not been titrated for these particular experiments, thus the optimal dose and time point for administration might not have been reached. The number of mice in these experiments are also limited. Depletion of CD25^+^ cells deplete not only Tregs but also T effector cells that might have impact on the results. An inbread mouse strain, such as C57BL/6 might give biased results, however the fact the role of IL2 and Tregs were very similar when both different mice strains (NMRI and C57BL/6) as well as different bacterial strains (LS-1 and NMRI) were used support our findings.

## Conclusions

IL2 in high doses is clinically approved as a cancer therapy because of the desired anti-tumor effects of Teffs. The same molecule in low doses preferentially expands and activates Tregs without affecting the Teffs and seems to represent a promising and safe treatment option in patients with various diseases where inflammation is thought to play a pivotal role, such as in various autoimmune diseases [21, 23, 44, 45], graft-versus-host disease [46] and atherosclerosis [47, 48]. In this study, IL2 gene delivery led to a sustained expansion of CD4^+^CD25^+^FoxP3^+^ Tregs, at the same time leaving Teffs unaffected, suggesting that the strategy of using rAAV-induced IL2 production probably resembles low-dose IL2 therapy in patients. To date there is insufficient evidence to judge whether low-dose IL2 therapy increases the susceptibility to infections in these patients. However, our results suggest that patients treated with low-dose IL2 may not be at an increased risk of hematogenously spread *S. aureus* arthritis, but on the contrary, an infection might have a milder course, and that the addition of low-dose IL2 to antibiotics in *S. aureus* infections might prove beneficial if provided very early.

## Data Availability

The datasets used and analyzed during the current study are available from the corresponding author on reasonable request.

## Abbreviations

CD: Cluster of differentiation
FoxP3: Forkhead box protein 3
GM-CSF: Granulocyte-macrophage colony-stimulating factor
gMFI: geometric mean fluorescence
IFN: Interferon
IL: Interleukin
NK: Natural Killer
NMRI: Naval Medical Research Institute
rAAV-IL2: recombinant adeno-associated virus vectors encoding for IL2
rAAV-LUC: recombinant adeno-associated virus vectors encoding for luciferase
RANKL: Receptor activator of nuclear factor kappa-Β ligand
rhIL2: recombinant human interleukin 2
*S. aureus*: *Staphylococcus aureus*
Teffs: T effector cells
Tregs: T regulatory cells
TSST-1: Toxic Shock Syndrome Toxin 1
Vg: Viral genomes
